# Pectin as Rheology Modifier of a Gelatin-Based Biomaterial Ink

**DOI:** 10.3390/ma14113109

**Published:** 2021-06-05

**Authors:** Anna Lapomarda, Elena Pulidori, Giorgia Cerqueni, Irene Chiesa, Matteo De Blasi, Mike Alexander Geven, Francesca Montemurro, Celia Duce, Monica Mattioli-Belmonte, Maria Rosaria Tiné, Giovanni Vozzi, Carmelo De Maria

**Affiliations:** 1 Research Center ‘E. Piaggio’, University of Pisa, via Diotisalvi, 1, 56122 Pisa, Italy; irene.chiesa@phd.unipi.it (I.C.); f.montemurro@centropiaggio.unipi.it (F.M.); g.vozzi@ing.unipi.it (G.V.); carmelo.demaria@unipi.it (C.D.M.); 2 Department of Ingegneria dell’Informazione, University of Pisa, via Girolamo Caruso, 16, 56122 Pisa, Italy; m.deblasi1@studenti.unipi.it; 3 Department of Chemistry and Industrial Chemistry, University of Pisa, via G. Moruzzi 13, 56124 Pisa, Italy; elena.pulidori@unipi.it (E.P.); celia.duce@unipi.it (C.D.); mariarosaria.tine@unipi.it (M.R.T.); 4 Department of Scienze Cliniche e Molecolari, Università Politecnica delle Marche, via Tronto 10/A, 60121 Ancona, Italy; g.cerqueni@pm.univpm.it (G.C.); m.mattioli@staff.univpm.it (M.M.-B.); 5 Laboratory of Polymers and Biomaterials, Istituto Italiano di Tecnologia, via Morego 30, 16163 Genova, Italy; mike.geven@iit.it

**Keywords:** 3D bioprinting, pectin, gelatin, biofabrication, extrusion-based bioprinting

## Abstract

Gelatin is a natural biopolymer extensively used for tissue engineering applications due to its similarities to the native extracellular matrix. However, the rheological properties of gelatin formulations are not ideal for extrusion-based bioprinting. In this work, we present an approach to improve gelatin bioprinting performances by using pectin as a rheology modifier of gelatin and (3-glycidyloxypropyl)trimethoxysilane (GPTMS) as a gelatin–pectin crosslinking agent. The preparation of gelatin–pectin formulations is initially optimized to obtain homogenous gelatin–pectin gels. Since the use of GPTMS requires a drying step to induce the completion of the crosslinking reaction, microporous gelatin–pectin–GPTMS sponges are produced through freeze-drying, and the intrinsic properties of gelatin–pectin–GPTMS networks (e.g., porosity, pore size, degree of swelling, compressive modulus, and cell adhesion) are investigated. Subsequently, rheological investigations together with bioprinting assessments demonstrate the key role of pectin in increasing the viscosity and the yield stress of low viscous gelatin solutions. Water stable, three-dimensional, and self-supporting gelatin–pectin–GPTMS scaffolds with interconnected micro- and macroporosity are successfully obtained by combining extrusion-based bioprinting and freeze-drying. The proposed biofabrication approach does not require any additional temperature controller to further modulate the rheological properties of gelatin solutions and it could furthermore be extended to improve the bioprintability of other biopolymers.

## 1. Introduction

Developing biocompatible and biodegradable biomaterial formulations with suitable rheological properties for the production of high-resolution tissue-like constructs remains one of the major challenges in extrusion-based bioprinting [1,2]. This powerful biofabrication technology enables the assembly of three-dimensional (3D) complex-shaped scaffolds by dispensing both cell-laden bioinks and acellular biomaterial inks in pre-designed locations [3,4]. These scaffolds provide a 3D template and structural support for cellular growth during new tissue formation.

An optimal biomaterial ink [4] should be readily extruded through the printing tip, forming continuous and homogenous fibers. Once extruded, fibers should (i) retain their shape without spreading over the deposition stage, (ii) not collapse on the previously extruded fibers, and (iii) provide structural support to the subsequently bioprinted layers. Additionally, the crosslinking approaches and the post-printing treatments, eventually required to further stabilize the scaffolds, should not alter the cytocompatibility of the biomaterial and the shape fidelity of the bioprinted scaffolds [1,2,5,6]. Shear-thinning pseudo plastic biomaterial formulations presenting a certain yield stress are the most promising candidates to be used as biomaterial inks, as these formulations behave as solids at low shear stresses and as fluids at higher shear stresses above the yield value. Furthermore, their viscosity decreases with increasing shear rates, allowing for facile extrusion [1,5,6,7].

During the last decades, a large number of biomaterial inks have been developed and characterized in the literature and are now commercially available (such as alginate [8], collagen [9], and gelatin methacrylate-based inks [10]). Among these, gelatin-based formulations play an important role in the fields of tissue engineering and regenerative medicine due to their excellent biocompatibility and biodegradability [11,12]. Gelatin is a water-soluble protein derived from the hydrolysis of collagen, a structural protein of the native extracellular matrix (ECM) [13]. Their chemical composition and ability to form hydrogels enable gelatin solutions to mimic the physical features and the biochemical processes typical of ECM [11,14]. In addition, the abundance of arginine–glycine–aspartic acid (RGD) sequences in the polymer backbone of gelatin promotes cell adhesion and proliferation [15,16]. The physical properties of gelatin solutions depend on several factors including source, temperature, pH, and gelatin concentration [17,18]. Pristine gelatin solutions generally behave as Newtonian fluids at higher temperatures (greater than ~30 °C) but form solid and stiff hydrogels at low temperature (20–25 °C) [18,19]. Therefore, their rheological properties (too rigid and viscous for extrusion below 20 °C and too fluid for structural integrity above 30 °C) make it very challenging for bioprinting self-sustaining scaffolds with an interconnected microporosity [20].

Several strategies have been employed so far to control the rheological properties of gelatin solutions before, during, and after the bioprinting process. These approaches are mainly based on the following: (i) incorporation of structural elements as rheology modifiers (e.g., nanoparticles [21] and biopolymers [22]) into the gelatin matrix; (ii) bioprinting gelatin into sacrificial support biomaterials [23]; and (iii) different chemical crosslinking approaches [24,25]. Together with these approaches, the bioprinter setup can be redesigned to further control the temperature of gelatin during extrusion (e.g., using a heated cartridge to affect the gel-to-sol transition of gelatin and facilitate its extrusion [26]) and/or after extrusion (e.g., using a cooled printing plate to induce gelation and improve shape fidelity [27]). Moreover, multiple printheads can be integrated, enabling the bioprinting of gelatin with other biopolymer inks as structural reinforcement for the gelatin solutions [28].

Here, we propose an approach to improve the extrusion-based bioprinting of gelatin-based solutions by use of pectin as rheology modifier and (3-glycidyloxypropyl)trimethoxysilane (GPTMS) as crosslinking agent for both the gelatin and pectin.

Pectin is a structural polysaccharide that occurs in soft tissues of land plants such as leaves, young shoots, and fruits [29,30]. Commercial pectin is mainly extracted from apple pomace and citrus peel and is commonly used as a gelling and thickening agent in the food processing industry [30,31]. Based on the degree of esterification (DE) of galacturonic acid units in its repeating structure, pectin can be classified as high methoxy (DE > 50%) and low methoxy (DE < 50%) content polymers [29]. For its gelling capabilities, biocompatibility, and biodegradability, pectin has already been employed for producing micro-beads for cell delivery and scaffolds for tissue regeneration obtained through freeze-drying [32], electrospinning [33], and 3D bioprinting [34,35]. In this study, we aim to exploit the thickening properties of pectin to enhance the bioprintability of low viscosity gelatin solutions for the first time. In particular, we hypothesize that the use of pectin as a rheology modifier of gelatin solutions may increase the viscosity and the yield stress of these formulations, rendering them suitable for extrusion-based bioprinting without the need for additional temperature controllers (such as cooling/heating devices), which would make the entire biofabrication process more complex. Additionally, the use of GPTMS as a single crosslinking agent for both pectin and gelatin may further simplify both the preparation process of gelatin–pectin biomaterial inks and the bioprinting process itself. 

With this aim, the preparation of homogenous gelatin–pectin formulations crosslinked with GPTMS was initially optimized. Freeze-dried gelatin–pectin–GPTMS sponges were initially produced, as our previous work demonstrated that the GPTMS crosslinking process requires a drying step to complete it [35]. These sponges were used to investigate the intrinsic properties of the microporous gelatin–pectin–GPTMS network in terms of porosity, pore size, degree of swelling, and compressive modulus. Furthermore, cell experiments using MG-63 osteoblast-like cells were carried out to analyze the effect of pectin content on cell adhesion. Finally, rheological and bioprinting assessments were performed using the gelatin–pectin–GPTMS formulations in order to assess whether they are suitable as biomaterial inks. Water stable, 3D woodpile scaffolds with micro- and macroporosity were successfully obtained by combining extrusion-based bioprinting and freeze-drying. In contrast, collapsed scaffolds with poor shape fidelity were obtained without the use of pectin. The results demonstrate the pivotal role of pectin in improving the bioprinting performance of gelatin solutions. Moreover, this facile biofabrication approach can potentially be extended to improve the bioprintability of other biopolymers that are otherwise challenging to process.

## 2. Materials and Methods

### 2.1. Materials

Low methoxy citrus pectin (Classic CU 701, DE 36%, MW 100−400 kDa [36]) was kindly provided by Herbstreit & Fox (Neuenburg, Germany). Gelatin (porcine Type A, gel strength 300 g bloom), GPTMS, hydrochloric acid (HCl), trypsin, ethylenediaminetetraacetic acid (EDTA), sodium cacodylate buffer, hexamethyldisilane, and ethanol were purchased from Sigma Aldrich (Saint Louis, MI, USA). Dulbecco’s phosphate-buffered saline (DPBS, P0750) without calcium and magnesium (ionic strength 170 mM) was acquired from Biowest (Meda, Italy). Glutaraldehyde and phosphate buffer solution (PBS) were supplied by Merck (Milano, Italy). Dulbecco’s modified Eagle’s medium (H-DMEM) and fetal bovine serum were acquired from Corning Inc (New York, NY, USA). The XTT assay kit and penicillin–streptomycin were acquired from Roche (Basel, Switzerland) and ThermoFisher (Waltham, MA, USA), respectively. Ultrapure water (Arium mini ultrapure lab water system, Sartorius, Varedo, Italy) was used in all experiments. Unless stated otherwise, all chemicals were used as received.

### 2.2. Preparation of GELPect Slurries and GELPectG Microporous Sponges

Homogenous slurries were obtained by mixing gelatin and pectin in DPBS (pH = 7.4) at 70 °C for 1 h at different ratios (Table 1). The pH of each slurry was thereafter lowered to 3.6 by drop-wise addition of 1 M HCl before the addition of GPTMS. Specifically, 368 µL and 920 µL [35] of GPTMS per gelatin and pectin gram were added, respectively.

Subsequently, the slurries were stirred for 40 min and poured into custom-made cylindrical molds (13 mm diameter × 10 mm height) to produce microporous sponges. The cylindrical GELPectG samples were initially frozen at −20 °C overnight and subsequently freeze-dried (BenchTop Pro-SP Scientific) at −60 °C for 24 h. The obtained microporous sponges were left on a heating plate at 40 °C for at least 4 days to promote the completion of the GPTMS reaction [35], and subsequently washed in ultrapure water to remove the traces of unreacted GPTMS. Each sponge was finally dried and used for further characterization.

### 2.3. Physical Characterization of GELPectG and Microporous Sponges

The effect of pectin content on the shape and architecture of pores of GELPectG sponges was investigated by scanning electron microscopy (SEM). GELPectG sponges (n = 3) were gold-sputtered by Edwards Sputter Coater B150S equipment (Edwards Distributor, Cinquepascal srl, Trezzano sul Naviglio Milan, Italy)) and observed with a Philips XL 20 SEM (FEI Italia SRL, Milan, Italy) microscope. Pore diameter was measured using at least 50 pores per SEM image (n = 3) by using ImageJ^®^. The porosity (%) of GELPectG sponges was calculated gravimetrically by applying Equation (1).

(1) $$Porosity=\left(1-\frac{m_{s}}{\rho_{s}V_{s}}\right)\;\times \;100$$

In Equation (1), *m_s_* and *V_s_* are the mass (g) and the volume (cm^3^) of each sponge (n = 3), respectively. Furthermore, *ρ_s_* is the theoretical density (g/cm^3^) of GELPect1G, GELPect2G, and GELPect3G, respectively. This parameter was calculated as the ratio of the mass and volume of disk-shaped samples (n = 10, Ø = 5 mm, 150 µm thick), which were die-cut from non-porous solvent-casted GELG and GELPect1G, and GELPect2G and GELPect3G films, respectively [35].

Degree of swelling at predefined timepoints was determined by soaking GELPectG sponges (n = 3) in ultrapure water at 37 °C. At each timepoint, the excess of water on the surface of GELPectG sponges was removed with tissue paper, and the weight of each sponge was thereafter measured. Equation (2) was used for the determination of degree of swelling (%).

(2) $$Degree\;of\;swelling=\frac{W_{s}-W_{0}}{W_{0}}\;\times \;100$$

In Equation (2), *W*_0_ and *W_s_* are the weight of sponges (g) before and after being soaked in ultrapure water, respectively. 

Finally, the effect of pectin on the mechanical properties of GELPectG sponges was investigated. Compressive stress–strain curves of dry and hydrated GELPectG sponges (n = 5 per composition and test condition) were determined by uniaxial compressive tests using a Z005 series Zwick/Roell testing (Genova, Italy) machine equipped with a 100 N load cell. Hydrated sponges were soaked in ultrapure water at 37 °C until the degree of swelling equilibrated. Prior to compressive tests, hydrated sponges were dabbed with tissue paper to remove the excess water. The compression rate was set to 0.01 s^−1^, and sponges were compressed up to 30% of their original heights. The compressive modulus was thereafter determined from the slope of the linear region of engineering stress–strain curves (at strain ranges from 0 to 10%). 

In all the experiments, GELG sponges were additionally tested as controls.

### 2.4. Biological Characterization of GELPectG Microporous Sponges

Human MG-63 osteoblast-like cells (ATCC, Manassas, VA, USA, CRL-1427) were cultured in H-DMEM containing 1% penicillin–streptomycin (PenStrep) and 10% fetal bovine serum (FBS) in a humidified incubator at 37 °C and 5% CO_2_. For passaging, trypsin/EDTA (trypsin 0.05%–EDTA 0.02% in PBS) was used. The medium was refreshed every 3 days. Before seeding, GELPectG and GELG sponges (Ø = 5 mm, thickness = 3 mm, n = 3) were sterilized in 70% ethanol for 20 min and thereafter under ultraviolet light for 30 min for each side. After sterilization, sponges were washed three times with PBS (pH 7.4) to neutralize the pH of GELPectG sponges. The sponges were subsequently preconditioned overnight (37 °C and 5% CO_2_) in 48 well plates containing complete H-DMEM without phenol red and supplemented with PenStrep and FBS, as indicated above. After removing the preconditioning medium, MG-63 cells were seeded on the sponges at a concentration of 2 × 10^4^ cells/sponge. The plates were incubated at 37 °C and 5% CO_2_. Cell viability, adhesion, and morphology were assayed at 48 h after cell seeding, as described below.

Cell viability of MG-63 was assessed by an XTT assay according to the manufacturer’s instructions. Briefly, the XTT labelling mixture was added to the medium in each well at a 1:2 ratio and incubated (for 4 h (37 °C, and 5% CO_2_). Then, the absorbance of the medium at 555 nm was quantified by spectrophotometry (MultiskanGo, Thermo Scientific, Monza, Italy) using a reference wavelength of 655 nm. Cell viability was calculated using Equation (3).

(3) $$Cell\;Viability\;\left(\%\right)=\frac{A_{GELPectG}}{A_{GELG}}\;\times \;100\;$$

In Equation (3), *A_GELPectG_* and *A_GELG_* are the absorbance at 555 nm of the medium incubated with seeded GELPectG and GELG sponges, respectively.

The influence of pectin content on cellular adhesion was evaluated at 48 h post seeding by microscopic observation using SEM. Prior to SEM analysis, seeded sponges (n = 2) were fixed in 0.1 M sodium cacodylate buffer containing 2% glutaraldehyde. The sponges were subsequently washed in 0.1 M cacodylate buffer containing 7% sucrose, followed by 0.1 M sodium cacodylate buffer containing 1% osmium tetroxide. Complete dehydration of seeded sponges was achieved in a graded ethanol series (25, 50, 70, 80, 95%, and 100%, respectively) and critical point drying was performed with hexamethyldisilane. After complete drying, sponges were sputter-coated with gold (Edwards Sputter Coater B150S, Edwards Distributor, Cinquepascal srl, Trezzano sul Naviglio Milan, Italy) and observed using a Philips XL 20 SEM (FEI Italia SRL, Milan, Italy) microscope.

### 2.5. Preparation and Rheological Investigation of GELPectG Biomaterial Inks

After the addition of GPTMS, GELPectG slurries were further stirred at 70 °C to promote the pre-crosslinking reaction of gelatin–pectin with GPTMS [35]. Once a visible increment of viscosity was observed, each slurry was considered bioprinting-ready. These formulations in the ‘ready-to-print’ state are hereafter referred to as biomaterial inks. 

Prior to bioprinting, rheological investigations were carried out. To isolate the contributions of pectin and GPTMS on the bioprintability of the formulations, both GELPect slurries (without GPTMS) and GELPectG biomaterial inks (with GPTMS) were tested separately. Likewise, GEL slurries and GELG biomaterial inks were further analyzed as controls. The experiments were performed at 25 °C (bioprinting temperature) on a HAAKE RheoStress 6000 (Thermo Scientific, Waltham, Massachusetts, USA) rheometer equipped with a Peltier temperature control unit. A cone−plate geometry with a diameter of 60 mm and a cone angle of 1° was used with a gap of 52 μm. 

Preliminary evaluation on the transition from liquid to solid-like behavior as a function of temperature was performed on GELPect slurries. This was determined from the cross-over point of the storage (G’) and loss (G’’) moduli during a temperature sweep from 15 to 40 °C (heating rate of 2.5 °C/min). All measurements were performed in the linear viscoelastic region (1 Hz, 0.1 Pa).

Viscosity curves were acquired at shear rates between 0.01 and 1000 s^−1^ to investigate the shear thinning behavior. Additionally, the yield stress of GELPect slurries and GELPectG biomaterial inks was calculated using the flow curves (shear rate γ vs. shear stress τ) and the tangent cross-over method [37]. This parameter is an indicator of shape retention after extrusion of the GELPect formulations with and without GPTMS.

### 2.6. Extrusion-Based 3D Bioprinting of GELPectG Biomaterial Inks

Before bioprinting, GELPectG biomaterial inks were loaded into a 5 mL syringe using a spatula and were cooled to room temperature (25 °C). Care was taken to prevent the entrapment of air bubbles during the transfer of the inks. A customized piston-driven extruder 3D bioprinter [23] (Supplementary Materials, Figure S1) was used for these experiments. The bioprintability of GELPectG biomaterial inks was initially evaluated through qualitative analysis. Each GELPectG biomaterial ink was dispensed (at room temperature) through a variety of extrusion tips to investigate their ability to form homogenous and uniform fibers rather than droplets [1]. Conical tapered plastic and straight tips of different diameters (ranging from 18 G to 25 G) were initially tested to identify the smallest possible extrusion tip that allowed for material extrusion without clogging, in order to obtain the highest possible resolution. A second assessment step was performed by testing the ability of GELPectG fibers to stack layer-by-layer to form 3D structures without collapsing. A G-code for woodpile scaffolds (15 × 15 × 3 mm^3^) with 1.66 mm spacing between fibers was written for this purpose. The presence of opened and interconnected lateral macropores in the xz and yz planes of the bioprinted structures (with z as the stacking direction) was used as an indicator for the absence of collapse. The G-code was continuously optimized during the bioprinting assessment by changing parameters such as the layer height. Moreover, the optimal printing parameters were found by tuning the flow rate from 250% and 450% with respect to the nominal flow needed to obtain a line with a width equal to the nozzle diameter. Printing speed was furthermore optimized between 6 mm/s to 15 mm/s. To exemplify these optimizations, when flattened fibers were obtained, the layer height was increased, while discontinuous fibers indicated that the flow rate needed to be raised or the printing speed to be lowered [1].

### 2.7. Statistical Analysis

Results are presented as mean ± standard deviation. A Student’s *t*-test was performed to compare properties of GEL or GELG (control) and properties of other GELPect slurries and GELPectG biomaterial inks, respectively. A minimum significance level of 0.05 was used in all the statistical tests performed. The symbol * was used in figures to indicate the significance level, as explained in figure captions.

## 3. Results and Discussion

### 3.1. Preparation of GELPect Slurries

**Figure 1:**
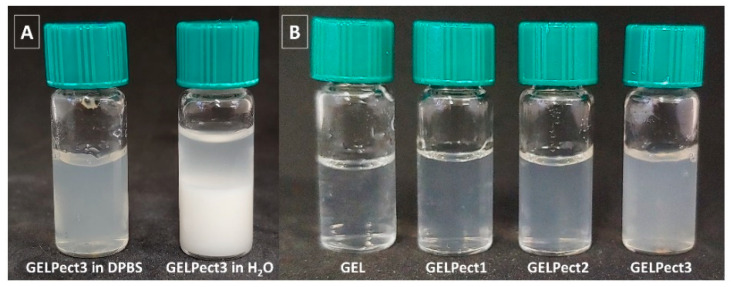
Representative picture of GELPect3 in DPBS and ultrapure water (**A**) and picture of GELPect slurries with different gelatin to pectin ratios in DPBS (**B**).

The preparation of GELPect slurries was initially optimized by visual assessments of slurry appearance and flow behavior. First attempts at preparing GELPect formulations were performed using ultrapure water. However, unstable and phase-separated slurries were obtained in this case (Figure 1A), due to the possible formation of insoluble gelatin–pectin complex coacervates [38,39]. Conversely, homogenous and viscous slurries were successfully produced with GELPect in DPBS (pH 7.4, ionic strength 170 mM) solvent for all pectin contents (Figure 1B). We hypothesize that the ionic strength of DPBS and its initial pH may have prevented the formation of gelatin–pectin aggregates. The presence of free ionic species in DPBS, in fact, may have weakened the electrostatic attraction between positively charged gelatin and the negatively charged pectin [38,39].

After 1h of mixing, the pH of each slurry decreased due to the presence of the acidic pectin, stabilizing at approximately 6.8, 5.5, 4.4, and 3.7 for GEL, GELPect1, GELPect2, and GELPect3, respectively. Subsequently, we investigated the addition of GTMS as crosslinking agent of both gelatin and pectin. GPTMS is necessary to prevent the dissolution of GELPect bioprinted scaffolds in an aqueous environment, such as cell culture medium, or in in vivo conditions. However, introduction of GPTMS to GELPect slurries initially caused a visible decrease of viscosity, thus reducing their bioprinting performances. Therefore, we lowered the pH of each slurry to 3.6 by the addition of aqueous HCl (1 M) before the addition of GPTMS (the pH of the slurries did not change after GPTMS addition). The previously-observed viscosity reduction was compensated by the decrease of pH to 3.6, however, which catalyzed the hydrolysis reaction of GPTMS [40] and resulted in an increased viscosity. 

The presence of siloxane network formation by GPTMS within the gelatin–pectin matrix was investigated by attenuated total reflectance infrared spectroscopy (ATR-IR) experiments (Figure S2, Supplementary Materials). The presence of the Si–O–Si band (~1100–940 cm^−1^) in all ATR-IR spectra of GELPectG formulations confirmed the successful crosslinking of gelatin–pectin by GPTMS.

### 3.2. Physical Characterization of GELPectG Microporous Sponges

**Figure 2:**
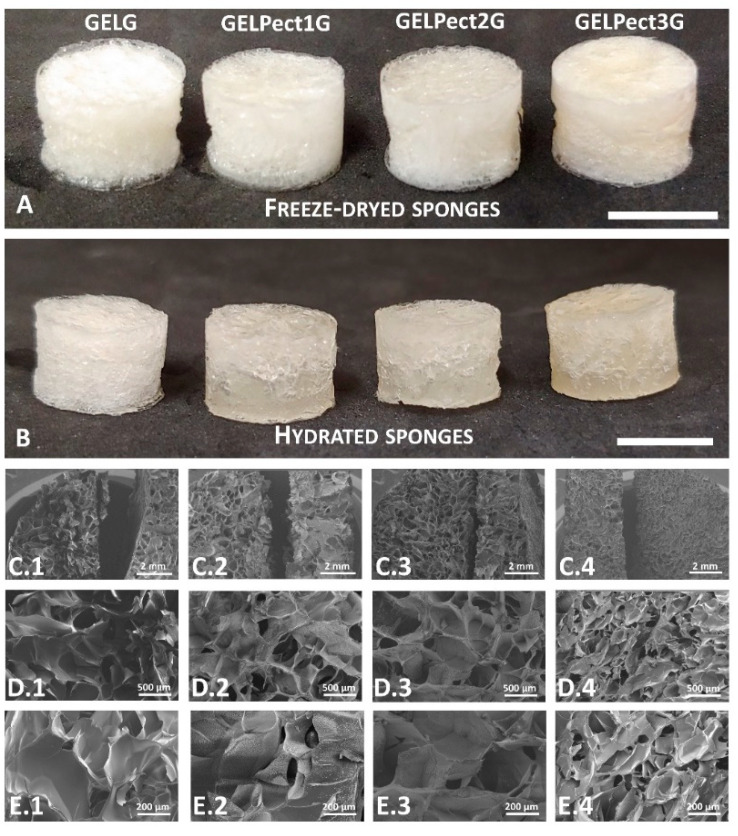
Images of GELG, GELPect1G, GELPect2G, and GELPect3G sponges as-freeze dried (**A**) and after rehydration (**B**); SEM images of freeze-dried GEL (**C.1**,**D.1**,**E.1**), GELPect1G (**C.2**,**D.2**,**E.2**), GELPect2G (**C.3**,**D.3**,**E.3**), and GELPect3G (**C.4**,**D.4**,**E.4**) sponges (scale bars: A, B = 10 mm, C.1–C.4 = 2 mm, D.1–D.4 = 500 µm, E.1–E.4 = 200 µm).

Freeze-dried microporous GELPectG sponges were initially prepared (Figure 2A,B) in order to investigate the intrinsic properties of the GELPectG networks in terms of pore size, porosity, degree of swelling, compressive modulus, and cell adhesion. As demonstrated in our previous work [35], the freeze-drying step is necessary to dry the scaffolds, and therewith drive the crosslinking reaction of GPTMS to completion. This step introduced micro-pores within the polymer matrix. The resulting increase in surface area to volume ratio of the sponges may be useful for cell migration and scaffold colonization [41,42]. 

The presence of micropores and the effect of pectin content on pore morphology and architecture were investigated by SEM analysis (Figure 2C.1–E.4).

All sponges possessed interconnected micropores with the typical foam-like architecture resulting from freeze-drying. The mean pore size was found to be 270 µm (with a range of 70–580 μm), 295 µm (range 118–588 μm), 305 µm (range 90–500 μm), and 118 µm (range 40–248 μm) for GELG, GELPect1G, GELPect2G, and GELPect3G sponges, respectively. The variations of the average and range of pore size might be a consequence of the cooling rate (from 25 °C to -–60 °C) used to prepare the microporous sponges. Although this parameter was carefully controlled during the fabrication of GELPect sponges, unpredictable temperature fluctuations may have affected the rate of ice-crystal nucleation and growth and therefore the size of the micropores [43]. Porosity of GELPectG sponges was determined gravimetrically and was found to be 89.1 ± 0.5, 88.9 ± 1.3, 88.5 ± 0.7, and 86.9 ± 0.4% for GELG, GELPect1G, GELPect2G, and GELPect3G, respectively. All the sponges showed values of porosity higher than 75%, which is the minimum value of porosity recommended for scaffolds with applications in tissue engineering. As a matter of fact, highly porous scaffolds with an interconnected pore network facilitate diffusion of nutrients (such as oxygen and glucose) and allow for metabolic waste removal [42].

**Figure 3:**
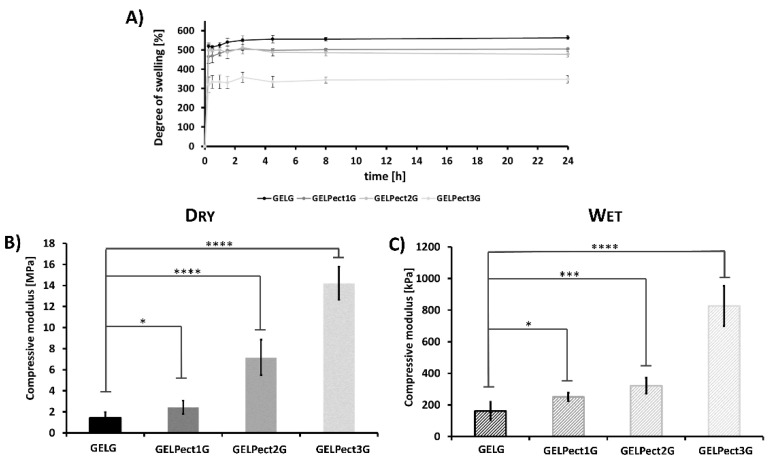
Degree of swelling over time of GEL, GELPect1, GELPect2, and GELPect3 sponges (**A**). Compressive modulus of dry (**B**) and wet (**C**) GELG, GELPect1G, GELPect2G, and GELPect3G sponges. (Student’s *t*-test significance levels * = *p* < 0.05, *** = *p* < 0.0005, **** = *p* < 0.0001, comparing the compressive modulus of GELG (control) and other GELPectG sponges).

Together with porosity and pore size, the degree of swelling in aqueous medium is another important parameter for tissue engineering scaffolds. The ability of a polymer matrix to entrap/absorb adequate amounts of water enables it to mimic the physical properties of the natural ECM. The degree of swelling of GELG and GELPectG sponges over time is shown in Figure 3A. GELG and GELPectG sponges demonstrated rapid water uptake after only 15 min of incubation in ultrapure water at 37 °C, reaching a plateau value at 8 h. At this timepoint, the degree of swelling was 555.9 ± 8.5, 500.8 ± 7.4, 486.3 ± 16.6, and 343.5 ± 14.5% for GELG, GELPect1G, GELPect2G, and GELPect3G, respectively. The introduction of pectin, and the consequent addition of GPTMS, clearly affected the water absorption of GELPectG sponges. Particularly, an increase of pectin and GPTMS content resulted in a decreasing degree of swelling. The increased GPTMS content (necessary for crosslinking both gelatin and pectin) may have enhanced the hydrophobicity of GELPectG sponges due to the increased amount of GPTMS siloxane chains [35,44].

Compressive moduli of dry and hydrated GELG and GELPectG sponges are presented in Figure 3B,C. Hydrated sponges were also tested to investigate the mechanical behavior of GELPectG sponges in in vitro-like conditions. Compared to dry sponges, the adsorption of water by GELG and GELPectG sponges resulted in a reduction of the stiffness. Globally, in both conditions, the compressive modulus of GELPectG sponges increased with pectin content and the relative GPTMS concentration. Particularly, the stiffness values recorded for the sponges with the highest pectin content (GELPect3G) were approximately 10 and 5 times higher than those of GELG sponges in dry (Figure 3B) and wet conditions (Figure 3C), respectively. As expected, the GPTMS-mediated crosslinking reaction resulted in higher stiffness gelatin–pectin matrices [35,45].

### 3.3. Biological Characterization of GELPectG Sponges

**Figure 4:**
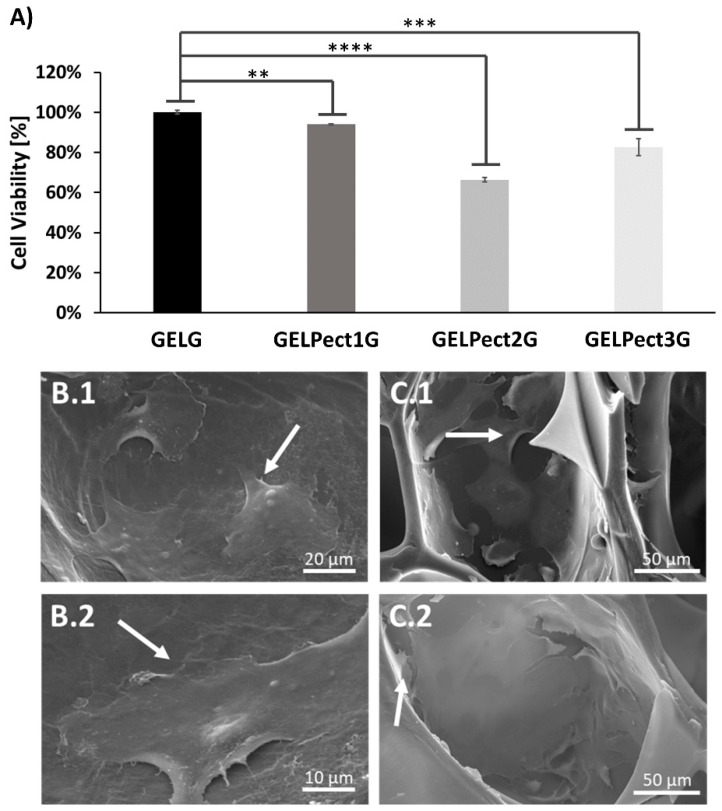
Cell viability at 48 h of MG-63 cells seeded on GELG and GELPectG sponges (Student’s *t*-test significance levels: ** = *p* < 0.005, *** = *p* < 0.0005, **** = *p* < 0.0001, comparing the metabolic activity of cells on GELG (control) and on other GELPectG sponges) (**A**). Representative SEM images of adherent MG-63 cells on GELPect1G (**B.1**,**B.2**), and GELPect3G (**C.1**,**C.2**) sponges at 48 h. (Scale bars: B.1 = 20 µm, B.2 = 10 µm, C.1,C.2 = 50 µm).

At 48 h, cell viability of MG-63 human osteoblast-like cells seeded on porous GELPectG sponges was evaluated to determine the effect of the pectin content on cell response. GELG sponges were used as control. Cell viability of MG-63 was tested by an XTT assay, and the results are shown in Figure 4A. As can be seen, a reduction of the metabolic activity of MG-63 cells of about 6% (GELPect1G), 34% (GELPect2G), and 17% (GELPect3G) was observed compared to GELG sponges. This reduction may be due to the increasing GPTMS content necessary to crosslink both gelatin and pectin. This results in decreased wettability of GELPect sponges and may delay cell adhesion, as already shown by Tonda-Turo et al. [44].

The presence and the morphology of adherent MG-63 cells on GELPectG and GELG sponges were further investigated by SEM analysis. Representative SEM images of adherent cells on GELPectG sponges are shown in Figure 4B.1,B.2,C.1,C.2. Adherent cells on GELPect1G and GELPect3G sponges exhibited elongated filopodia, a flattened cytoplasm, and a star-like shape typical of this cell line. Notably, adherent MG-63 cells were also found inside the pores of GELPect3G sponges (Figure 4C.1,C.2).

Although preliminary results from the XTT assay demonstrated a slight decrease of metabolic activity of MG-63 cells on GELPectG compared to GELG sponges, the presence of adherent cells inside the micropore network of GELPect sponges at 48 h indicated that GELPectG sponges were not cytotoxic. They provided a suitable surface for cell attachment, and pectin incorporation did not negatively affect the cytocompatibility of gelatin.

### 3.4. Extrusion-Based Bioprinting of GELPectG Biomaterial Inks

#### 3.4.1. Preparation of GELPectG Biomaterial Inks and Rheological Investigation

The effect of pectin inclusion and GPTMS crosslinking on the bioprintability of gelatin slurries at room temperature (25 °C) was evaluated by rheological experiments.

**Figure 5:**
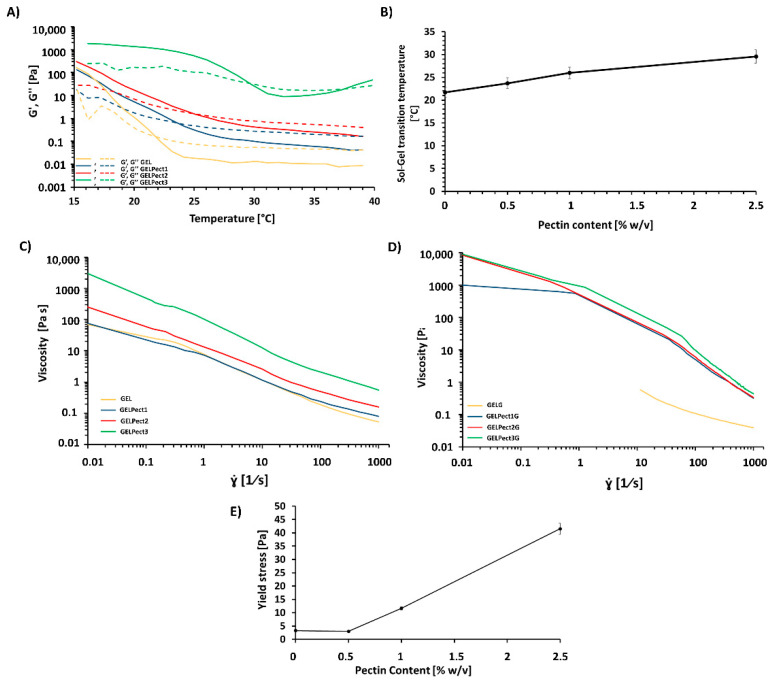
Temperature dependence of storage modulus (G’, continuous line) and loss modulus (G’’, dashed line) of GEL and GELPect slurries within the linear viscoelastic region (1 Hz, 0.1 Pa) (**A**); sol–gel transition temperature of GELPect slurries as a function of pectin concentration (**B**); experimental viscosity curves (**C**,**D**) at 25 °C of GEL and GELPect slurries (**C**), and of GELG and GELPectG biomaterial inks (**D**), and yield stress of GELPect slurries as a function of pectin content at 25 °C (**E**).

The effect of pectin content on the transition between the solid and liquid state of GELPect slurries (without GPTMS) was initially investigated through temperature sweep tests (ranging from 15 °C to 40 °C) in the linear viscoelastic region (1 Hz, 0.1 Pa). The gel-to-sol transition temperature was identified as the intersection of G’ and G’’ (Figure 5A). As demonstrated in Figure 5B, this parameter increased with pectin content (from approximately 21.7 °C to 29.6 °C for GEL and GELPect3 slurries, respectively), which is in agreement with other works in the literature [46]. The gel-to-sol temperature is an important parameter for bioprinting. Liquid-like biomaterial formulations are not suitable for extrusion-based bioprinting, as they do not retain their shape once extruded and tend to flow out over time. The increment in gel-to-sol transition temperature demonstrated that pectin made gelatin a gel-like slurry at room temperature (25 °C), even in the absence of a crosslinker, thus avoiding the need for any additional temperature control system. 

Following GPTMS addition, GELPectG and GELG formulations were further stirred at 70 °C to induce an increment in viscosity useful for extrusion-based bioprinting [35]. As already mentioned, the introduction of GPTMS caused an initial decrease of viscosity of GELPect and GEL slurries in DPBS if their pH was not adjusted. Reduction of pH to 3.6 (by 1 M HCl addition) together with the stirring at 70 °C accelerated the pre-crosslinking reaction between GPTMS and gelatin–pectin [40]. This resulted in more viscous GELPectG formulations. The time required to obtain such an increase of viscosity depended on both pectin and GPTMS content (see Table 2). Increasing pectin and GPTMS contents resulted in a more rapid increment in viscosity. 

The viscosity curves of GELPect slurries and GELPectG biomaterial inks are shown in Figure 5C,D, respectively. All the formulations demonstrated a shear thinning behavior, which is beneficial for extrusion-based bioprinting. The low viscosity under shear allows for facile extrusion through the extruder tip, while the high viscosity when no shear stress is applied (at rest conditions) may prevent excessive material flow on the bioprinting stage after extrusion [47].

To investigate the effect of pectin and/or GPTMS on the ‘at rest’ behavior of GELPect slurries and GELPectG biomaterial inks, their yield stresses were determined (Table 3). As can be seen in Figure 5E, increasing pectin contents in GELPect slurries results in higher values of yield stress. The pre-crosslinking reaction of GPTMS in the GELPectG biomaterial inks resulted in a further increase of the yield stress due to the Si–O–Si network formation within the gelatin–pectin matrix (Table 3).

The GEL slurry and the GELG biomaterial ink, deemed not-printable, showed significantly lower viscosities and negligible values of yield stress compared to GELPect slurries and GELPectG biomaterial inks, respectively. 

Combined, these results demonstrate the pivotal role of pectin in modulating the rheological properties of gelatin biomaterial inks, as was also shown for cellulose inks by Cernencu et al. [34] (pectin–nanocellulose ink formulations). Although pectin alone already modulates the rheological properties of GELPect slurries, the presence of a crosslinker agent is necessary to crosslink both gelatin and pectin, which would otherwise dissolve under physiological conditions. Notably, a synergistic effect of pectin incorporation and the GPTMS pre-crosslinking reaction on GELPectG rheological properties was observed, rendering these inks suitable for bioprinting applications. 

#### 3.4.2. Bioprinting Assessment of GELPectG Biomaterial Inks

**Figure 6:**
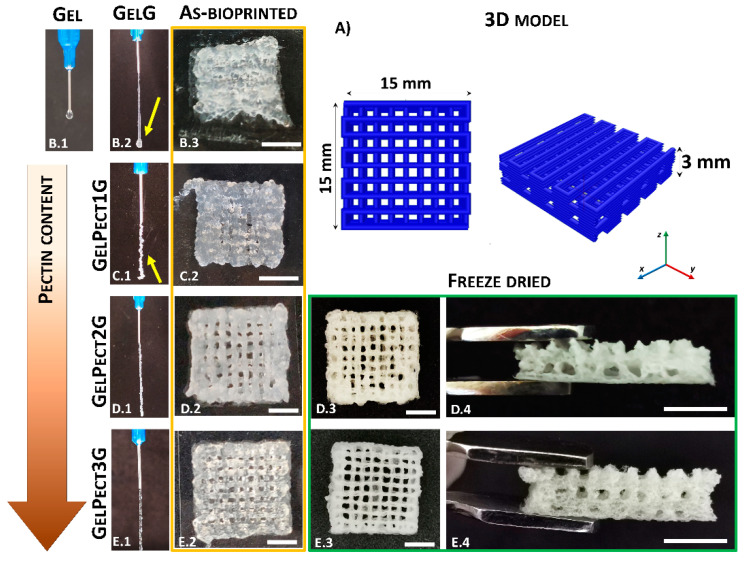
Illustration of the 3D model of a woodpile scaffold (**A**). Images of the initial bioprinting screening to evaluate fiber formation of GEL (no GPTMS) (**B.1**), GELG (**B.2**), GELPect1G (**C.1**), GELPect2G (**D.1**), and GELPect3G (**E.1**); and pictures of 3D woodpile structures as-bioprinted (**B.3**,**C.2**,**D.2, E.2**) and freeze-dried (**D.3**,**D.4**,**E.3**,**E.4**) (scale bars = 5 mm).

Bioprinting assessments were carried out to confirm the capability of GELPectG biomaterial inks to form self-sustaining scaffolds with high shape fidelity at ambient conditions (25 °C). Initial bioprinting investigation focused on the formation of homogenous and continuous fibers by dispensing inks through extrusion tips with varying shapes (tapered and straight) and diameters (18 G–25 G). A 22 G straight tip was identified as the optimal extrusion tip, as it was the smallest tip through which it was possible to extrude all the formulations (with the maximum extrusion force achievable by the bioprinter used in this work: 1570 N [48]). As shown in Figure 6B.2,C.1, GELG and GELPect1G formulations did not form homogenous filaments when extruded, while continuous and uniform fibers were produced with both GELPect2G (Figure 6D.1) and GELPect3G (Figure 6E.1). The inability to bioprint the GEL slurry (without GPTMS) was further confirmed by the formation of drops rather than fibers when extruded at 25 °C (Figure 6B.1). 

The bioprinting process was subsequently optimized by identifying the optimal flow rate and bioprinting speed to produce self-sustaining woodpile structures with interconnected macropores (Figure 6A). The optimal values of flow rates and bioprinting speeds together with all the bioprinting parameters used in this study are summarized in Table 2. 

Collapsed woodpile structures with poor shape fidelity were obtained for all tested flow rates and bioprinting speeds with GELG (Figure 6B.3) and GELPect1G (Figure 6C.2) biomaterial inks. For GelG, this is a direct result of the lack of filament formation together with the low viscosity and negligible values of yield stress. Once extruded, the GELG biomaterial ink did not maintain shape and tended to spread on the bioprinting plate, collapsing over the previously-printed struts over time. Similar results were obtained with the GELPect1G biomaterial ink, although the presence of pectin did reduce the spreading of this biomaterial formulation, due to an increase of yield stress (Table 3). Conversely, self-standing woodpile structures with open and interconnected macropores and good shape fidelity were obtained with both GELPect2G (Figure 6D.2–D.4) and GELPect3G (Figure 6E.2–E.4). This result can be explained by the higher yield stress of these two formulations (Table 3) compared to GELG and GELPectG1 biomaterial ink. This prevented excessive flow of GELPect2G and GELPect3G after extrusion, enhancing their shape retention once bioprinted. Woodpile scaffolds with the highest shape fidelity were obtained by GELPect3G biomaterial inks. In addition, woodpile structures with different numbers of layers (7, 12, and 18) were successfully bioprinted (Figure S4, Supplementary Materials) to demonstrate the capability of this formulation to sustain a large number of printed layers without collapsing.

Once bioprinted, GELPect2G and GELPect3G woodpile scaffolds were freeze-dried to complete the GPTMS crosslinking reaction (Figure 6D.3,D.4,E.3,E.4). This step introduced micro-pores within the 3D bioprinted struts, increasing their surface area. Subsequently, the scaffolds were rehydrated in ultrapure water at 37 °C to assess their shape retention in wet conditions. It was found that the freeze-drying and the rehydration steps did not negatively alter the shape of GELPect2G and GELPect3G scaffolds (Figure S5, Supplementary Materials).

Finally, to further demonstrate the unique role of pectin on gelatin bioprintability, woodpile scaffolds were bioprinted using GELPect slurries (without GPTMS) (Figure S6, Supplementary Materials). Even though these slurries are bioprintable and an increasing pectin content enhances bioprintability, the absence of a crosslinking agent would not allow for their use in the production of scaffolds for biological investigations. The physiological conditions (e.g., a hydrated environment at 37 °C) would cause the complete dissolution of the bioprinted scaffolds in a short time. It is nonetheless interesting that pectin alone renders gelatin slurries bioprintable.

## 4. Conclusions

Gelatin is a natural biopolymer widely used in tissue engineering applications, since it mimics the biophysical properties of natural ECM. However, its rheological properties limit its application as biomaterial ink for extrusion-based 3D bioprinting. This study addresses this challenge by using pectin as a gelatin thickening agent, and GPTMS as a gelatin and pectin crosslinker. The first part of the study was focused on developing bioprintable gelatin–pectin formulations by tuning the pectin content. Homogenous and transparent gelatin–pectin formulations crosslinked with GPTMS were successfully obtained using DPBS as solvent. Subsequently, the effects of pectin on the properties of freeze-dried sponges crosslinked with GPTMS were studied. Results from this initial investigation showed that microporous sponges with interconnected micro-pores were obtained at all pectin concentrations. Porosity and degree of swelling decreased with pectin content, while sponge stiffness increased with pectin content both in dry and wet conditions. Cell experiments showed that pectin did not negatively alter the biocompatibility of gelatin, and that MG-63 cells adhered on the surface and started to grow within the micropores of GELPect3G sponges after 48 h. Finally, the effect of pectin content on the gelatin bioprintability was assessed. Results from a rheological investigation revealed that pectin plays a key role in improving gelatin bioprintability as it causes an increase of viscosity and yield stress. Moreover, its addition allows for bioprinting at ambient conditions (25 °C) without the employment of any additional temperature controller. The viscosity and yield stress further increased when GPTMS pre-crosslinking of the slurries was performed. The presence of GPTMS as crosslinking agent prevents the dissolution of gelatin and pectin in physiological conditions. Self-standing woodpile scaffolds with interconnected macro- and microporosity were successfully obtained at the highest pectin content (GELPect2G and GELPect3G, respectively) by combining 3D bioprinting and freeze-drying. These results show the great potential of using pectin as a thickening agent for gelatin in the bioprinting field for the first time. Moreover, the use of a single crosslinking agent for both gelatin and pectin allows the preparation of the biomaterial inks and the bioprinting process to be strongly simplified. This avoids post-printing crosslinking treatments (such as incubation in a crosslinking medium), which may negatively affect the shape fidelity of the bioprinted scaffolds.

Knowledge of the effect of pectin on the bioprintability of other natural hydrogels, such as collagen or hyaluronic acid, can expand its application as a rheology modifier for extrusion-based bioprinting applications.

## Figures and Tables

**Table 1:** Composition of gelatin-pectin formulations in DPBS.

Gelatin (% *w*/*v*)	Pectin (% *w*/*v*)	Name without GPTMS/Name with GPTMS
5	0	GEL/GELG
5	0.5	GELPect1/GELPect1G
5	1	GELPect2/GELPect2G
5	2.5	GELPect3/GELPect3G

**Table 2:** Bioprinting parameters used to bioprint GELG, GELPect1G, GELPect2G, and GELPect3G biomaterial inks.

Bioprinting Parameters	GELG	GELPect1G	GELPect2G	GELPect3G
Time required to induce increase of viscosity before printing (h)	>24	>10	8	5
Printing temperature (°C)	25	25	25	25
Type of tip	Straight	Straight	Straight	Straight
Size of the tip (G)	22	22	22	22
Layer height (µm)	250	250	250	250
Extrusion multiplier (%) ^a)^	320	380	400	440
Printing speed (mm/s)	4	4	5	6

^a)^ Value set in the slicing software (Slic3r^®^), defined as a percentage of the theoretical flow rate needed to obtain a line width equal to the nozzle size according to the software.

**Table 3:** Yield stress values of GELPect slurries and GELPectG biomaterial inks. (The yield stress of GELG was not detectable).

	No GPTMS				With GPTMS			
Parameters	GEL	GELPect1	GELPect2	GELPect3	GELG	GELPect1G	GELPect2G	GELPect3G
Yield Stress (Pa)	3.3	3.0	11.6	41.5	-	48.2	474.5	489.8

## Data Availability

No publicly accessible data are available for this work.

## Supplementary Materials

The following are available online at https://www.mdpi.com/article/10.3390/ma14113109/s1, Figure S1: Picture of the extrusion-based bioprinter used in this work, Figure S2: Representative picture of GELPect3 composition in DPBS and ultrapure water, Figure S3: ATR-IR spectra of uncrosslinked GELPect films and crosslinked GELPectG films by GPTMS, Figure S4: pictures of 3D bioprinted GELPect3G woodpile scaffolds with different numbers of layers, Figure S5: optical microscopy images of 3D bioprinted GELPect2G and GELPect3G scaffolds after bioprinting, freeze-drying and rehydration, Figure S6: representative pictures of 3D bioprinted scaffolds made of GEL, GELPect1, GELPect2 and GELPect3 slurries (without GPTMS).
