# Supplementary material for: Pectin as Rheology Modifier of a Gelatin-Based Biomaterial Ink
Source: Materials (Basel). 2021 Jun 5;14(11):3109. doi: 10.3390/ma14113109 (PMC8201283; doi:10.3390/ma14113109)
Supplement: Supplementary file 1 [file materials-14-03109-s001.zip › materials-1231219-supplementary.pdf]

Supplementary materials

Pectin as Rheology Modifier of a Gelatin-Based Biomaterial Ink

Anna Lapomarda*, Elena Pulidori, Giorgia Cerqueni, Irene Chiesa, Matteo De Blasi, Mike A. Geven, Francesca Montemurro, Celia Duce, Monica Mattioli Belmonte, Maria R. Tiné, Giovanni Vozzi and Carmelo De Maria

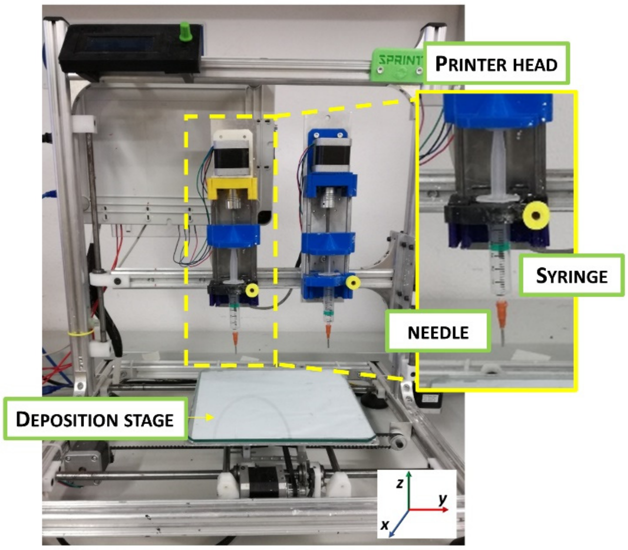

Figure S1. Picture of the extrusion-based bioprinter used in this study. Insert: printer head made of a 5 mL disposable syringe and a needle through which the biomaterial formulations are extruded on the deposition stage.

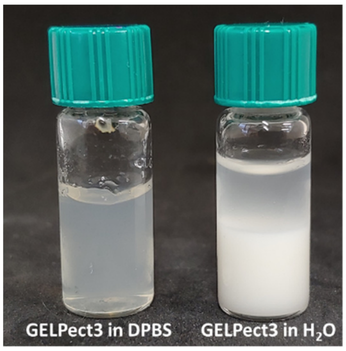

Figure S2. Representative picture of GELPect3 composition in DPBS and ultrapure water. The acidic pH (~3) of gelatin-pectin aqueous slurries resulted in a strong electrostatic attraction between gelatin and pectin, which may have caused the formation of large insoluble gelatin-pectin complexes. The large size of these aggregates made their dispersion unsustainable, and therefore they tend to precipitate overtime.

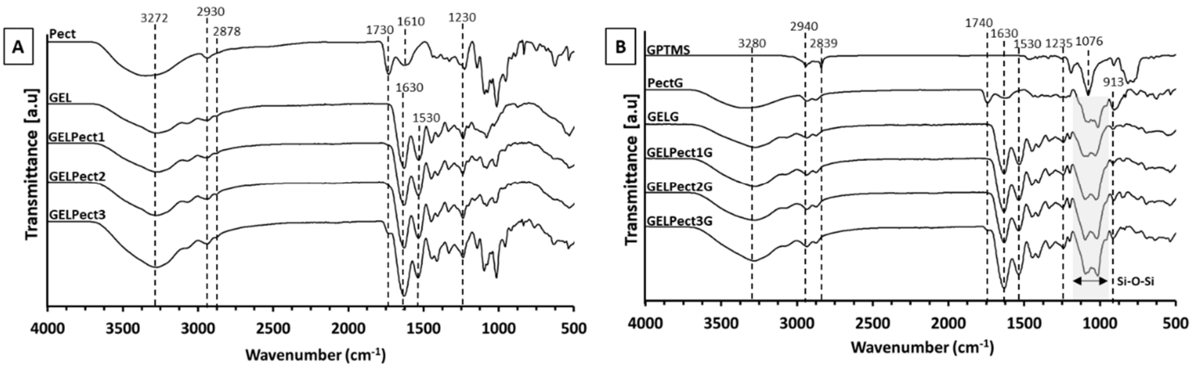

Figure S3. ATR-IR spectra of uncrosslinked pectin (Pect), gelatin (GEL), GELPect1, GELPect2 and GELPect3 (A), and of raw GPTMS, crosslinked pectin (PectG), gelatin (GELG), GELPect1G, GELPect2G and GELPect3G (B).

As shown in Figure S3.A, the ATR-IR spectrum of pectin displays the characteristic peaks at 2930 cm^{-1}, 1730 cm^{-1} and 1610 cm^{-1} due to $-CH_2$ and $-CH_3$ stretching vibrations (2930 cm^{-1}), the stretching vibrations of the methyl ester groups ($-COOCH_3$) and the undissociated carboxyl acid groups (-COOH) (1730 cm^{-1}), and to the asymmetric stretching vibration of the carbonyl group of the carboxylate ion ($-COO^-$) (1610 cm^{-1})[1]. The ATR-IR spectrum of gelatin showed a broad band at 3272 cm^{-1} corresponding to the stretching vibrations of -OH and -NH groups, and peaks at 2932 cm^{-1} and 2878 cm^{-1} due to CH_2 symmetrical and asymmetrical stretch. Furthermore, it showed the characteristic peaks at 1630 cm^{-1} (amide I, C=O stretching), 1530 cm^{-1} (amide II, N-H bending) and 1230 cm^{-1} (amide III, C-N stretching) [2]. In the ATR-IR spectra of GELPect films the carbonyl peak at 1730 cm^{-1} appeared with increasing pectin contents. The presence of a gelatin-pectin complex formation should be detected within the region 1600- 1530 [3,4], however, the increased content of $-COO^-$ pectin groups produces a peak in this region and therefore gelatin-pectin complexation could not be delineated.

As shown in Figure S2.B, in the ATR-IR spectra of GPTMS the peaks at 2940 cm^{-1} and 2839 cm^{-1} were attributed to $-CH_2$ and $-CH_3$ symmetric and asymmetric stretch, respectively; and a peak at 1076 cm^{-1} corresponding to the epoxy group. [5] The formation of GPTMS-based crosslinks in GELPectG, PectG and GELG films is evident from the appearance of Si-O-Si (~ 1100-940 cm^{-1}) band and Si-OH (~ 913 cm^{-1}) peak [6]. This confirmed the successful crosslinking of gelatin-pectin by GPTMS.

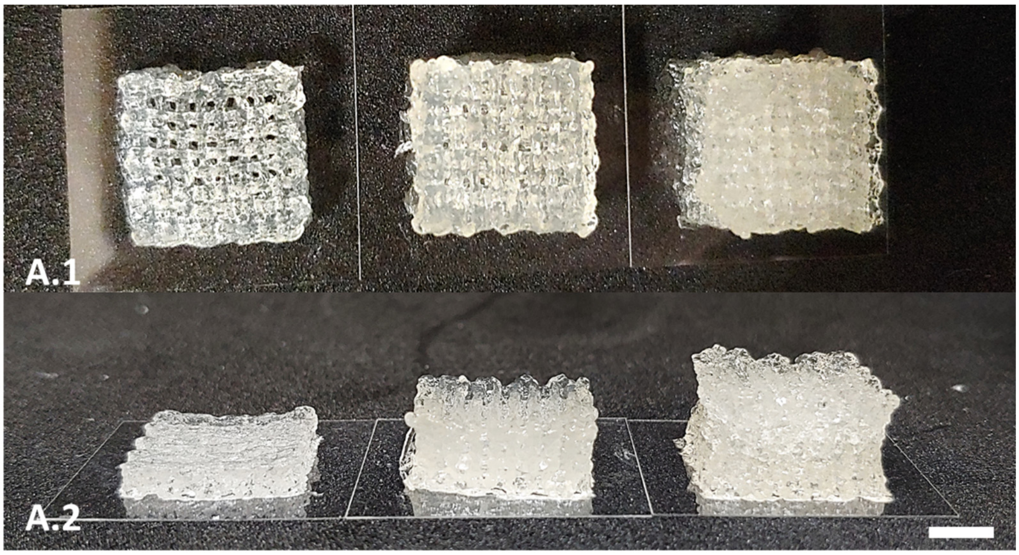

Figure S4. Images of top (A.1) and lateral views (A.2) of GELPect3G woodpile structures with 7 layers (left side structure in each image), 12 layers (middle structure in each image) and 18 layers (right side in each image) (scale bar = 5 mm).

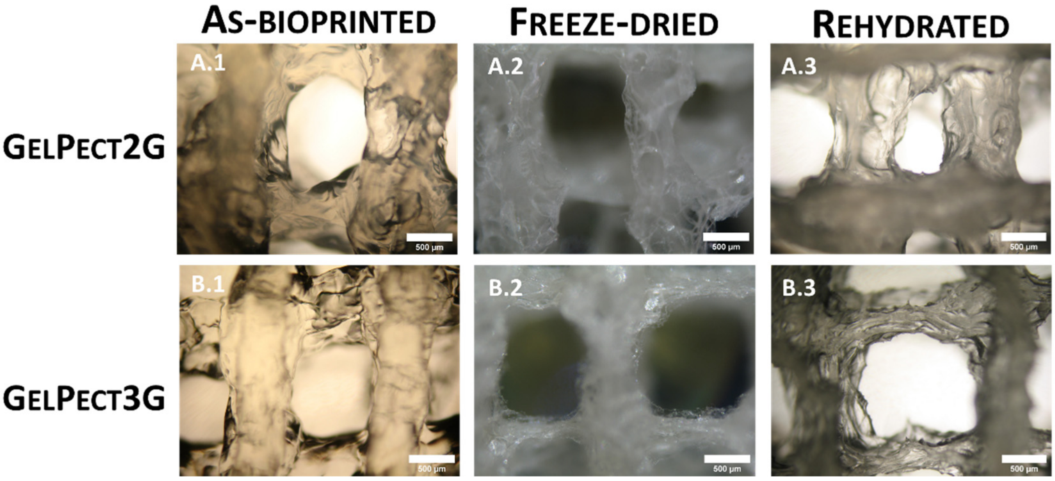

Figure S5. Optical microscopy images with top view of GELPect2G (A.1 - A.3) and GELPect3G (B.1 - B.3 woodpile structures after bioprinting (A.1, B.1), after freeze-drying (A.2, B.2) and after rehydration (A.3, B.3) (Scale bars = 500 μm).

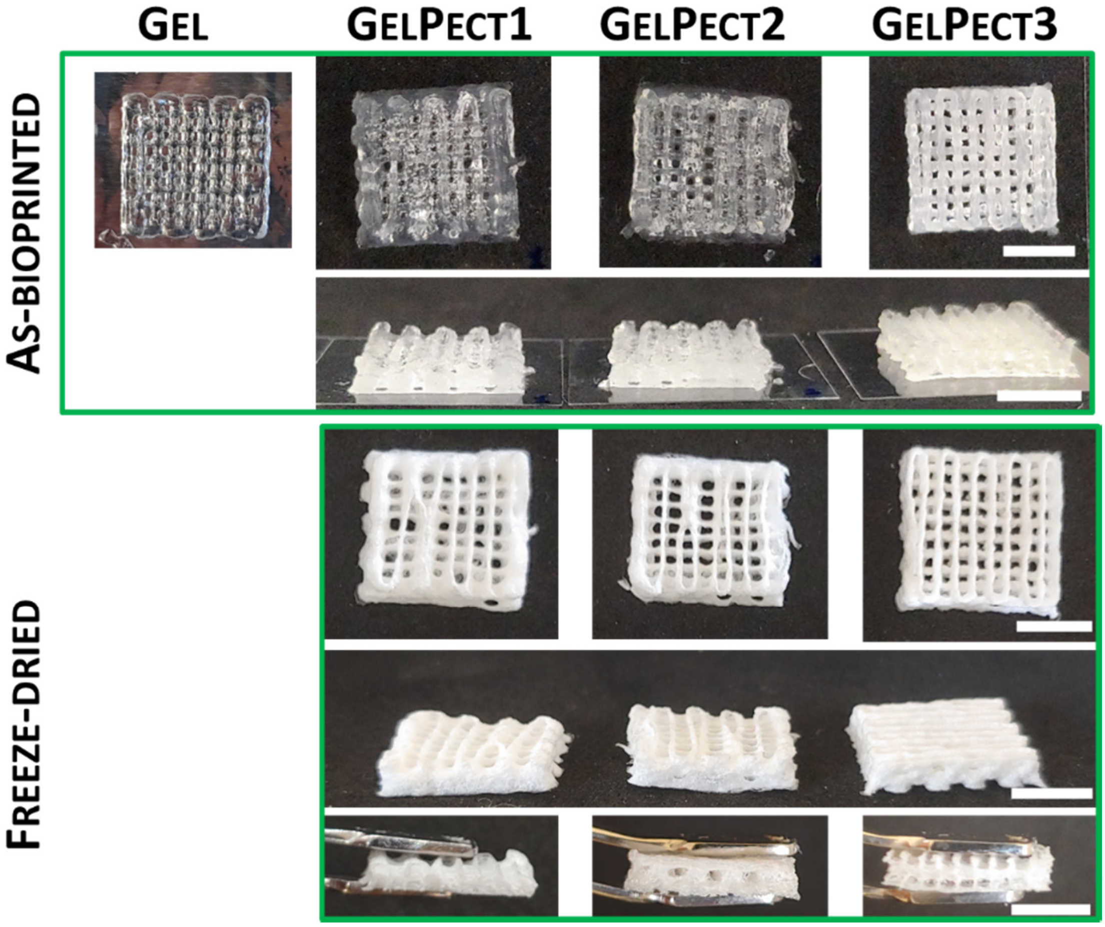

Figure S6. Top view and lateral view of 3D woodpile structures (15 × 15 × 3 mm^3) made of GEL, GELPect1, GELPect2 and GELPect3 slurries (no GPTMS) after bioprinting and freeze drying (Scale bar = 7.5 mm).

References

1. V. B. V. Maciel, C. M. P. Yoshida, and T. T. Franco, "Chitosan/pectin polyelectrolyte complex as a pH indicator," Carbohydr. Polym., vol. 132, pp. 537–545, 2015, doi: 10.1016/j.carbpol.2015.06.047.
2. J. H. Muyonga, C. G. B. Cole, and K. G. Duodu, "Fourier transform infrared (FTIR) spectroscopic study of acid soluble collagen and gelatin from skins and bones of young and adult Nile perch (Lates niloticus)," Food Chem., vol. 86, no. 3, pp. 325–332, 2004, doi: 10.1016/j.foodchem.2003.09.038.

3. B. Muhoza, S. Xia, J. Cai, X. Zhang, E. Duhoranimana, and J. Su, "Gelatin and pectin complex coacervates as carriers for cinnamaldehyde: Effect of pectin esterification degree on coacervate formation, and enhanced thermal stability," Food Hydrocoll., vol. 87, no. August 2018, pp. 712–722, 2019, doi: 10.1016/j.foodhyd.2018.08.051.
4. M. Saravanan and K. P. Rao, "Pectin-gelatin and alginate-gelatin complex coacervation for controlled drug delivery: Influence of anionic polysaccharides and drugs being encapsulated on physicochemical properties of microcapsules," Carbohydr. Polym., vol. 80, no. 3, pp. 808–816, 2010, doi: 10.1016/j.carbpol.2009.12.036.
5. Y. Shirosaki et al., "Physical, chemical and in vitro biological profile of chitosan hybrid membrane as a function of organosiloxane concentration," Acta Biomater., vol. 5, no. 1, pp. 346–355, 2009, doi: 10.1016/j.actbio.2008.07.022.
6. C. Tonda-Turo et al., "Comparative analysis of gelatin scaffolds crosslinked by genipin and silane coupling agent," Int. J. Biol. Macromol., vol. 49, no. 4, pp. 700–706, 2011, doi: 10.1016/j.ijbiomac.2011.07.002.
7. A. Lapomarda et al., "Pectin-GPTMS-Based Biomaterial: Toward a Sustainable Bioprinting of 3D scaffolds for Tissue Engineering Application," Biomacromolecules, vol. 21, no. 2, 2020, doi: 10.1021/acs.biomac.9b01332.
